# Stretch, Injury and Inflammation Markers Evaluation to Predict Clinical Outcomes After Implantable Cardioverter Defibrillator Therapy in Heart Failure Patients With Metabolic Syndrome

**DOI:** 10.3389/fphys.2018.00758

**Published:** 2018-06-26

**Authors:** Celestino Sardu, Raffaele Marfella, Matteo Santamaria, Stefano Papini, Quintino Parisi, Cosimo Sacra, Daniele Colaprete, Giuseppe Paolisso, Maria R. Rizzo, Michelangela Barbieri

**Affiliations:** ^1^Department of Medical, Surgical, Neurological, Metabolic and Aging Sciences, University of Campania “Luigi Vanvitelli”, Naples, Italy; ^2^Department of Cardiovascular and Arrhythmias, John Paul II Research and Care Foundation, Campobasso, Italy

**Keywords:** ST2 protein, heart failure, internal cardioverter defibrillator, ICDs' shocks, hospitalization

## Abstract

**Background:** Internal cardioverter defibrillator (ICD) therapy reduced all-cause mortality. Conversely, few studies reported that ICDs' shocks may reduce survival. Recently authors suggested that, multiple inflammatory and molecular pathways were related to worse prognosis in metabolic syndrome (MS) patients treated by ICDs. Therefore, it may be relevant to find new biomarkers to predict ICDs' shock and worse prognosis in treated patients.

**Methods:** In 99 MS vs. 107 no MS patients treated by ICD for primary prevention, we evaluated all-cause mortality, cardiac deaths, hospitalization for heart failure, appropriate and inappropriate therapy, and survival after appropriate ICD therapy.

**Results:** MS vs. no MS patients had higher levels of failing heart stress biomarkers. The highest values of ST2 were related to worse prognosis. Patients who had better survival after appropriate ICD therapy were those associated with lowest ST2 values. At multivariate Cox regression analysis, C reactive protein (CRP) (0.110 [0.027–0.446], *p*-value 0.002), troponine I (TnI) protein (0.010 [0.001–0.051], *p*-value 0.010), and B type natriuretic peptide (BNP) (1.151 [1.010–1.510], *p*-value 0.001), predicted all cause of deaths. BNP predicted cardiac deaths (1.010 [1.001–1.206], *p*-value 0.033). MS, and BNP predicted hospitalization for heart failure events (2.902 [1.345–4.795], *p*-value 0.001; 1.005 [1.000–1.016], *p*-value 0.007). ST2 predicted appropriate therapy (1.012 [1.007–1.260], *p*-value 0.001), as BNP (1.005 [1.001–1.160], *p*-value 0.028), LVEF (1.902 [1.857–1.950], *p*-value 0.001), and CRP (1.833 [1.878–1.993], *p*-value 0.028). ST2, and BNP predicted survival after ICD appropriate therapy (4.297 [1.985–9.302], *p*-value 0.001; 1.210 [1.072–1.685], *p*-value 0.024).

**Conclusions:** ST2 values may differentiate MS patients with a higher risk of ICDs' therapy, and worse prognosis. Therefore, ST2 protein may be used as valid monitoring biomarker, and as a predictive biomarker in failing heart ICDs' patients affected by MS.

## Introduction

Few years ago authors reported that, in a population of 1,232 post-ischemic patients with a left ventricle ejection fraction (LVEF) ≤30%, the implantation of internal cardioverter defibrillator (ICD) for primary prevention therapy brought to a 31% reduction of all-cause mortality (Moss et al., 2002). Subsequently, ICD was introduced as primary preventive, and save life treatment for patients with LVEF <35% due to prior myocardial infarction, and/or with non-ischemic dilated cardiomiopathy, in New York Heart Association (NYHA) functional class II-III (Kusumoto et al., 2014). Indeed, the ICD therapy prevented cardiac arrest, and sudden cardiac deaths (SCD) events, by performing anti-tachycardia pacing therapy (ATP), and shock therapy to arrest ventricular arrhythmias (VT), and ventricular fibrillations (VF) (Kusumoto et al., 2014). More in detail, in this case ATP and ICDs' shocks were named as appropriate interventions (Kusumoto et al., 2014). On the contrary, ICDs' intervention to terminate rapid and sustained atrial or supraventricular arrhythmias, were named as inappropriate ICDs' therapy (Kusumoto et al., 2014). Recently, a great interest has been invested to study clinical outcomes in patients, that experienced appropriate and/or inappropriate ICD's therapy, or both therapies (Proietti et al., 2015). In fact, as first the occurrence of ICDs' shocks has been associated to worse prognosis (Proietti et al., 2015). Secondary, this risk appeared to be greater for appropriate than inappropriate shocks (Proietti et al., 2015). Moreover, both appropriate and inappropriate therapies were associated with impaired survival (Proietti et al., 2015). Therefore, a growing interest has been focused to find new methods, and treatments to reduce, and/or to prevent appropriate, and inappropriate ICDs' therapies. In the clinical practice this may be translated in the opportunity to avoid the subsequent cardiac pump failure observed in patients after ICD therapy, and the related worse clinical prognosis, such as augmented hospitalizations, and deaths events (Proietti et al., 2015). Multiple hypothesis may explain the reason of pump failure in ICD patients. In this setting, a central role may be played by the hyper activation of numerous and different cardiac adaptive molecular pathways, with the consequent related structural alterations. In the past, cardiac myocyte injury biomarkers, systemic inflammatory markers, and myocardial stretch peptides, has been evaluated to predict deaths or appropriate ICDs' therapies, and to identify in overall population the patients' potential for survival benefit from ICDs' therapies (Scott et al., 2011). Intriguingly, this study was conducted in a population represented for 24% by diabetics, and for 75% by patients with LVEF <35% (Scott et al., 2011). Recently, authors showed that, diabetes, hypertension, overweight, and other risk factors leading together to the Metabolic Syndrome (MS), influenced in patients with LVEF <35% the ICDs' functionality, and heart failure disease progression by a pro-arrhythmic status (Sardu et al., 2017). In fact, this pro-arrhythmic status favoring ICDs' therapies, then can result in a reduction of the survival in MS patients (Sardu et al., 2017). Moreover, our study hypothesis was that, MS may condition a pro-arrhythmic status, and the rate of ICDs' therapies in patients with LVEF <35%. On other hand, it is not well known if myocyte injury biomarkers, systemic inflammatory markers, and myocardial stretch peptides serum values may predict ICD therapies, and survival in MS patients. Conversely, no studies reported data about these biomarkers assay to identify MS patients' potential for survival benefit from ICD therapy. Therefore, the study aim was to investigate all-cause mortality, cardiac deaths, hospitalization for heart failure, appropriate, and inappropriate ICDs' therapies, and survival rate with appropriate ICD therapy in a population of failing heart MS patients treated by ICD. However, we correlated these study outcomes to these biomarkers at baseline, and during follow up to stage MS patients at higher risk for ICDs' interventions, and mortality events, and to stratify the appropriateness of decision making as ICD implantation in failing heart MS patients. Moreover, in these patients we evaluated for any appropriate and inappropriate ICD delivered therapy, whether shock or ATP, hospital admissions for heart failure, and mortality events during a 1-year follow-up period.

## Methods

In a multicenter study, conducted from January 2014 to January 2017 at University of Campania, Luigi Vanvitelli, Naples (Italy), John Paul II Research and Care Foundation, Campobasso (Italy), and Catholic University of Sacred Heart, Campobasso, Italy, we screened a population of consecutive patients affected by ischemic cardiomyopathy, with severe reduction of LVEF (LVEF <35%), without prior ventricular arrhythmic event. These patients were referred to our clinic, underwent ICD implantation for primary prevention of SCD according to international guidelines (Priori et al., 2015), and entered in the study. However, these patients did not have a true left bundle branch block.

### Inclusion criteria were

Aged >18 and <75 years, both genders, NYHA functional class II to III, left LVEF <35%, stable coronary artery disease, and stable heart failure with indication to receive an ICD.

### Exclusion criteria were

Left bundle branch block, active myocarditis, unstable coronary artery disease, unstable heart failure, co-morbidities which may limit life to <6 months, history of cardiac surgery or intervention within the preceding 90 days, history of moderate to severe chronic obstructive pulmonary disease (COPD), defined as needing chronic oxygen therapy, or recent (within 30 days) hospitalization for COPD flare-up, pregnancy, and history of primary pulmonary hypertension; previous ICD, CRT-d and/or pacemaker implantation, absence of informed patient consent.

### Study protocol

Study population was then divided in MS patients, and controls patients (no MS patients). In short MS was diagnosed by the presence of three or more risk factors as obesity, dyslipidemia, hypertension, and insulin resistance (Sardu et al., 2017). Controls patients did not respect these diagnostic criteria, and were named as no MS patients. In all these patients an ICD was inserted for the first time according to guidelines suggestions (Priori et al., 2015). The trans thoracic echocardiographic exam was performed to assess left ventricular end-diastolic/systolic diameter, end-diastolic/systolic volumes, and LVEF. Before performing an ICD implantation all patients underwent coronary angiography. Before interventions, baseline laboratory studies, including HbA1c, lipid panel, fibrinogen, and renal function were determined. In this study population we measured by peripheral blood HF inflammation biomarkers, as C Reactive Protein (CRP) and Interleukine 6 (IL-6), HF Myocyte Injury biomarkers, as Cardiac-specific troponins I (CTnI) and Creatine kinase-MB (CK-MB), and HF Myocyte Stress biomarkers, as Brain natriuretic peptide (BNP), and ST2 protein.

The study was conducted in accordance with the Declaration of Helsinki. The protocol was approved by the Ethics Committees of all participating institutions.

### Study endpoints

As study endpoints, we monitored all cause deaths, cardiac deaths, hospitalizations for heart failure worsening, appropriate ICDs' therapy, inappropriate ICDs' therapy, and survival after appropriate ICDs' therapy. These study outcomes were assessed in MS vs. no MS patients, and in different ST2 quartiles, according to previously described methods (Scott et al., 2011; Pascual-Figal and Januzzi, 2015).

### Serum biomarkers analysis

We measured HF inflammation biomarkers (CRP and IL-6), HF Myocyte Injury biomarkers (CTnI, and CK-MB), HF Myocyte Stress biomarkers (BNP, and ST2 protein), neutrophiles and lymphocytes values, and their ratio (NLr) by peripheral blood samples. These biomarkers were measured to indicate their correlation to ICDs' interventions, hospitalizations, and mortality after ICDs' therapies (Scott et al., 2011). All these biomarkers, such as ST2 blood values, were measured at baseline, and during 6 and 12 months of follow up after ICD implantation. Blood samples from all centers were shipped to the central laboratory at John Paul II Research and Care Foundation. ST2 evaluation was performed by using Presage ST2 (Critical Diagnostics, San Diego, CA, USA), a quantitative monoclonal ELISA assay in a 96-well plate (ELISA PLATE READER DAS S.R.L; model A3 and C, Rome, Italy). In each well, we added 100 μL of blood serum. The lower limit of detection of ST2 was 2 ng/ml and the upper limit was 200 ng/ml. Normal sST2 values for patients in good health were <25 ng/mL (Ky et al., 2011), and indicated as first (I) ST2 quartile (ST2 value <25 ng/ml). Therefore, we indicated ST2 >25 and <50 ng/ml as second (II) ST2 quartile, ST2 >50 and <75 ng/ml as third (III) ST2 quartile, and ST2 >75 ng/ml as fourth (IV) ST2 quartile.

### Cardiac intervention

Experienced electrophysiologists in ICD implantation performed under local anesthesia, and by subclavian vein puncture, the positioning and implantation of the ICDs' catheters in the cardiac chambers. These catheters were connected to the ICD generator, then allocated in the subcutaneous subclavian space, ipsilateral to the subclavian vein puncture. All ICD implantation procedures were standardized. Right atrial catheters were all placed in right atrial appendage, and right ventricular catheters in right ventricle apex, as indicated by antero-posterior, right anterior, and left anterior oblique views projections at radioscopic imaging.

### Arrhythmic events, and ICDs therapies

Arrhythmic events were defined as atrial fibrillation (AF), ventricular tachycardia (VT), and ventricular fibrillation (VF) episodes. AF was defined as paroxysmal, and/or not paroxysmal according to authors' suggestions (January et al., 2014). VT was defined as arrhythmia originating from ventricular chambers, with a regular rhythm/cycle length, and evidence of ventricular fusion beats, and of ventricular-atrial dissociation (Priori et al., 2015). VT was defined as sustained and/or not sustained by arrhythmic event duration (Priori et al., 2015). VF was defined as a fibrillating arrhythmia originating from ventricular chambers, and associated to hemodynamic instability, and cardiac arrest (Priori et al., 2015). Similarly, by ICDs' devices interrogations at follow up we reported ICDs shocks for each ICD patient. ICDs' therapies were defined as anti-tachycardia pacing (ATP), ICD shocks, ATP plus ICD shocks, and not appropriate ICD shocks (Maisel, 2004). ICD shocks were defined as ICD induced high energy therapies to interrupt sustained VT and/or VF (Maisel, 2004). Appropriate ICD therapy was defined as ATP for VT, and shock therapy for VT or VF (Maisel, 2004). All the rest of ICDs' therapies were defined as inappropriate (Maisel, 2004). We monitored and collected these after implantation, according to previously described criteria (Maisel, 2004).

### Follow-up

Follow-up was concluded at 10th day, 6th month, and at 12th month after ICD implantation.

Follow-up visits were scheduled by the treating physician, and enrolled patients were followed by clinical visits, ecg, echocardiography, and device telemetric control. During these visits and device interrogations, arrhythmic events, and subsequently ICDs' interventions, and its effect in terms of clinical outcomes (hospitalizations for HF worsening, cardiac deaths, and all cause deaths) were reported. In addition, all patients were instructed to report about devices alarms, and arrhythmias, and they were instructed regularly to assess body weight, occurrence of dyspnea, and any clinical symptom. At each visit, patients were asked whether medical events or symptoms suggestive of cardiac arrhythmias occurred, and an ECG, and an ECG Holter monitoring, were performed to detect the presence of asymptomatic arrhythmias. At each visit, NYHA classification was re-assessed, and patients graded their overall condition as unchanged or slightly, moderately, or markedly worsened, or improved since randomization by global self-assessment (Sardu et al., 2016). Clinical evaluations included physical examination, vital signs, and review of adverse events. A fasting blood (at least 12 h from last meal) was performed for glycemia, lipid profile (total cholesterol (TC), triglycerides, HDL-C, and LDL-C), at every visit. BNP, ST2, IL-6, CRP, TnI, CKMB, leucocytes, and neutrophiles (and NLr), were evaluated.

### Statistical methods

All data were analyzed by two different physicians, and the patients divided before in MS group and no MS group (control group), and during follow-up visits and controls in patients with different ST2 protein values, by quartiles as indicated before in the text. We postulated that the number of patients with alterations in primary and secondary study endpoints was significantly different between MS patients and no MS patients, and between patients stratified in ST2 quartiles. The physicians were blinded to ST2 protein values at enrollment. We postulated that, the number of patients with primary and secondary study endpoints was significantly different between the first, second, third, and fourth ST2 quartiles. For this reason we defined at baseline a cut off of 25 ng/ml, to differentiate the lower ST2 group (first quartile) vs. the second ST2 quartile (25 <ST2<50 ng/ml), vs. the third ST2 quartile (50<ST2<75 ng/ml), vs. the higher ST2 group (fourth quartile, ST2>75 ng/ml). Safety analyses were performed on data from all enrolled patients. Continuous variables were presented as mean and standard deviation if normally distributed; otherwise, they were presented as median and inter-quartile range. Categorical variables were expressed as number and frequencies. Continuous variables were compared with an unpaired Student *t*-test, and categorical variables were compared using the Chi-square test or Fisher exact test as appropriate. Predictors of the primary study endpoint were evaluated by using Cox regression models in which covariates for the adjustment were selected if associated with a *p* < 0.25 at univariate analysis. A stepwise method with backward elimination was used and hazard ratios (HRs) with 95% confidence intervals (CIs) were derived. We considered a 2-sided *p*-value of < 0.05 as statistically significant. The statistical analysis was performed using the SPSS software package for Windows 17.0 (SPSS Inc., Chicago, IL).

## Results

Clinical characteristics of study population (n 206 patients) were reported in Table 1. MS patients (n 99) vs. controls (no MS, n 107) exhibited a higher percentage of diabetes [62 (62.6%) vs. 33 (30.8%), *p*-value 0.005], overweight [BMI >30, 66 (66.6%) vs. 31 (29%) patients, *p*-value 0.002), and ischemic heart failure (76 (76.8%) vs. 59 (55.1%) patients, *p*-value 0.047]. About patients affected by ischemic heart failure, the 77.6% (n 59) of MS patients and 83% (n 49) of controls patients had a previous myocardial infarction. Regards the pathophysiology of disorders of intraventricular conduction, the 24.2% (n 24) of MS patients, and 26.2% (n 28) of controls patients had right bundle branch block. The 20.2% (n 20) of MS patients and 21.5% (n 23) of controls patients had no specific intraventricular conduction delay. To date, 7.1% (n 7) of MS patients and 7.5% (n 8) of controls patients had a combination of right bundle branch block plus non-specific intraventricular conduction delay. Therefore, higher prevalence of MS vs. no MS patients were under ACE inhibitors [37 (37.4) vs. 19 (17.8), *p*-value 0.043], and ARS blockers [39 (39.4) vs. 23 (21.5), *p*-value 0.033]. Table 1. Similarly, in MS patients there was a higher percentage of insulin therapy [31 (31.3%) vs. 10 (9.3%), *p*-value 0.041], and oral hypoglycemic drug therapy [57 (57.6%) vs. 31 (29%), *p*-value 0.022]. Table 1. MS patients had higher serum levels of BNP (630.78 ± 50.25 vs. 487.01 ± 42.47 mmol/l, *p*-value 0.001), ST2 protein (134.12 ± 5.7 vs. 82 ± 7.85 ng/ml, *p*-value 0.001), and CRP (9. 16 ± 0. 94 vs. 7.35 ± 0.69 mg/l, *p*-value 0.05). Table 1. At follow up (6th and 12th month), MS patients vs. no MS patients still had higher levels of failing heart stress biomarkers (BNP at 6th month 182.95 ± 37.51 vs. 107.14 ± 33.25 mmol/l, *p*-value 0.0035; BNP at 12th month 175.41 ± 34.21 vs. 103.22 ± 31.25 mmol/l, *p*-value 0.004; ST2 protein at 6th month 114.12 ± 5.1 vs. 57 ± 7.32 ng/ml, *p*-value 0.031; ST2 at 12th month 111.38 ± 5.3 vs. 53 ± 7.12 ng/ml, *p*-value 0.004). Table 2.

**Table 1:** Baseline characteristics of study population.

**Parameters**	***Overall population (n 206)***	***MS (n 99)***	***No MS (n 107)***	***P-value***
Age years (mean + SD)	71 ± 9	70 ± 7	72 ± 8	n.s.
Male n (%)	145 (70.2)	69 (69.7)	76 (71)	n.s.
NYHA class II	45 (21.8)	24 (24.2)	21 (19.6)	n.s.
NYHA class III	160 (77.7)	75 (75.8)	85 (79.4)	n.s.
QRS duration msec	146.5 ± 11	145 ± 11	148 ± 11	n.s.
**RISK FACTORS**				
Hypertension	116 (56.3)	54 (54.5)	62 (57.9)	n.s.
Diabetes	99 (48)	62 (62.6)	33 (30.8)	0.005^*^
BMI>30	97 (47)	66 (66.6)	31 (29)	0.002^*^
Smokers	108 (52.4)	49 (49.5)	59 (55.1)	n.s.
Dyslipidemia	110 (53.4)	52 (52.5)	58 (54.2)	n.s.
Hypertension	161 (78.2)	78 (79.2)	83 (77.6)	n.s.
Ischemic heart failure	135 (65.5)	76 (76.8)	59 (55.1)	0.047^*^
COPD	36 (17.5)	16 (16.2)	20 (18.7)	n.s.
**IMPLANTED DEVICE**				
ICD-VVI	66 (32)	27 (27.3)	30 (28)	n.s.
ICD-DDD	140 (68)	72 (72.7)	77 (72)	n.s.
**MEDICATIONS AT BASELINE**				
Amiodarone	43 (20.8)	20 (20.2)	23 (21.5)	n.s.
Aspirin	83 (40.3)	41 (41.4)	42 (39.2)	n.s.
ACE inhibitors	56 (27.2)	37 (37.4)	19 (17.8)	0.043^*^
ARS blockers	62 (30)	39 (39.4)	23 (21.5)	0.033^*^
Sacubitril/valsartan	52 (25.2)	24 (24.2)	28 (26.2)	n.s
**Beta blockers**				
Carvedilol	63 (30.6)	28 (28.3)	35 (32.7)	n.s.
Bisoprolol	82 (39.8)	37 (37.4)	45 (42)	n.s.
Warfarin	73 (35.4)	32 (32.3)	41 (38.3)	n.s.
NOAC	41 (19.9)	22 (22.2)	19 (17.8)	n.s.
Tiklopidine	4 (1.9)	2 (2)	2 (1.8)	n.s.
Calcium antagonist	7 (3.4)	3 (3)	4 (3.7)	n.s.
Ivabradine	61 (29.6)	30 (30.3)	31 (29)	n.s.
Digoxin	65 (31.5)	30 (30.3)	35 (32.7)	n.s.
Loop diuretics	190 (92.2)	91 (91.9)	99 (92.5)	n.s.
Aldosterone Blockers	146 (64.6)	76 (76.8)	70 (65.4)	n.s.
Statins	147 (71.4)	70 (70.7)	77 (72)	n.s.
Insulin	41 (19.9)	31 (31.3)	10 (9.3)	0.041^*^
Oral Hipoglicemic drugs	88 (42.7)	57 (57.6)	31 (29)	0.022^*^
**ECHOCARDIOGRAPHIC PARAMETERS**				
LVEF	27.4 ± 5.4	27.4 + 5.7	28.2 ± 5.1	n.s.
LVEDd	67 ± 8	69 ± 6	66 ± 9	n.s.
LVESd	43 ± 7	42 ± 6	44 ± 8	n.s.
LVEDv	197 ± 39	194 ± 29	200 ± 48	n.s.
LVESv	135 ± 28	133 ± 21	138 ± 35	n.s.
**MITRAL INSUFFICIENCY**				
+	103 (50)	45 (45.4)	53 (49.2)	n.s.
++	80 (38.8)	38 (38.4)	42 (39.3)	n.s.
+++	26 (12.6)	15 (15.2)	12 (11.5)	n.s.
**BIOMARKERS**				
LYMPHOCYTES	7.92 ± 2.13	7.92 ± 2.12	7.62 ± 2.36	n.s.
NEUTROPHILES	5.31 ± 1.81	5.32 ± 1.80	5.26 ± 2.07	n.s.
IL-6	25.15 ± 1.9	26.08 ± 2.93	25.14 ± 2.79	n.s.
CRP	8.25 ±0.81	9.16 ±0.94	7.35 ±0.69	0.05^*^
BNP	487.01 ±28.9	630.78 ±50.25	487.01 ±42.47	0.001^*^
ST2	82 ±5.34	134.12 ±5.7	82 ±7.85	0.001^*^
TnI	0.15 ±0.0005	0.152 ±0.007	0.154 ±0.007	n.s.
CKMB	1.25 ±0.15	1.252 ±0.22	1.247 ±0.21	n.s.

In this table we have reported at baseline clinical characteristics, drug therapy, and echocardiographic parameters comparing Metabolic Syndrome (MS) patients v/s overall population (no MS). ACE is for angiotensin converting enzyme; ARS is for angiotensin receptros; BMI is for body mass index; BNP is B type natriuretic peptide; CKMB is cretin kinase MB fraction; COPD is chronic obstructive pulmonary disease; CRP is C reactive protein; ICD-VVI is for single chamber internal cardioverter defibrillator; ICD-DDD is for dual chamber internal cardioverter defibrillator; IL-6 is interleukin 6; y is for year; in mitral insufficiency the simble + is for low grade of reflow, ++ for moderate grade of reflow, +++ is more than moderate; LVEDd is for left ventricle diastolic diameter; LVESd is for left ventricle systolic diameter; LVEDv is for left ventricle end diastolic volume; LVESv is for left ventricle end sistolic volume; LVEF is for left ventricle ejection fraction; n is for number; NYHA is for New York Hearth Association; NOAC is for new oral anticoagulant; SD is for standard deviation; ST2 is protein ST2; TnI is troponine I. Statistical analysis has been conducted to compare categorical data with the exact Pearson's χ2 test. We considered a two-sided p-value of < 0.05 as statistically significant. A p-value < 0.05 has been marked with ^*^ symbol. A p > 0.05 is named as n.s.

**Table 2:** Study population in metabolic syndrome (MS) patients at 6 months and 12 months of follow up.

**Parameters**	***MS (n 99) at 6th months***	***No MS patients (n 107) at 6 months***	***P-value***	***MS (n 99) at 12th months***	***No MS patients (n 107) at 12 months***	***P-value***
NYHA class I	11 (11)	14 (13)	n.s.	10 (10)	15 (14)	n.s.
NYHA class II	21 (22)	28 (26)	n.s.	22 (22)	31 (29)	n.s.
NYHA class III	55 (55)	55 (52)	n.s.	53 (54)	52 (49)	n.s.
NYHA class VI	12 (12)	10 (9)	n.s.	14 (14)	9 (8)	n.s.
QRS duration msec	125 ± 9	128 ± 7	n.s.	124 ± 8	126 ± 8	n.s.
**IMPLANTED DEVICE**						
ICD-VVI	27 (27.3)	30 (28)	n.s.	/	/	
ICD-DDD	72 (72.7)	77 (72)	n.s.	/	/	
**MEDICATIONS AT FOLLOW UP**						
Amiodarone	29 (29.3)	26 (24.3)	n.s.	/	/	
Aspirin	44 (44.4)	45 (42)	n.s.	/	/	
ACE inhibitors	32 (32.3)	19 (17.8)	0.05^*^	/	/	
ARS blockers	37 (37.4)	23 (21.5)	0.043^*^	/	/	
Sacubitril/valsartan	35 (35.4)	36 (33.6)	n.s	/	/	
**Beta blockers**						
Carvedilol	32 (32.3)	39 (36.4)	n.s.	36 (36.4)	40 (37.4)	n.s.
Bisoprolol	39 (39.4)	46 (43)	n.s.	38 (38.4)	47 (43.9)	n.s.
Warfarin	34 (34.3)	40 (37.4)	n.s.	41 (41.4)	42 (39.2)	n.s.
NOAc	24 (24.2)	20 (18.7)	n.s.	24 (24.2)	20 (18.7)	n.s.
Tiklopidine	2 (2)	2 (1.8)	n.s.	2 (2)	2 (1.8)	n.s.
Calcium antagonists	2 (2)	2 (2.8)	n.s.	2 (2)	2 (2.8)	n.s.
Ivabradine	31 (31.3)	31 (29)	n.s.	31 (31.3)	31 (29)	n.s.
Digoxin	32 (32.3)	35 (32.7)	n.s.	30 (30.3)	31 (29)	n.s.
Loop diuretics	91 (91.9)	97 (90.6)	n.s.	92 (92.9)	95 (88.8)	n.s.
Aldosterone Blockers	79 (79.8)	74 (69.1)	n.s.	80 (80.8)	76 (71)	n.s.
Statins	70 (70.7)	77 (72)	n.s.	70 (70.7)	77 (72)	n.s.
Insulin	31 (31.3)	10 (9.3)	0.041^*^	/	**/**	
Oral Hipoglicemic drugs	57 (57.6)	31 (29)	0.022^*^	/	**/**	
**ECHOCARDIOGRAPHIC PARAMETERS**						
LVEF	33 ± 5	34 ± 4	n.s.	34 ± 6	36 ± 4	n.s.
LVEDd	62 ± 5	62 ± 4	n.s.	62 ± 5	60 ± 3	n.s.
LVESd	40 ± 5	41 ± 7	n.s.	39 ± 5	39 ± 7	n.s.
LVEDv	153 ± 22	146 ± 49	n.s.	151 ± 24	143 ± 47	n.s.
LVESv	115 ± 19	108 ± 33	n.s.	112 ± 20	105 ± 31	n.s.
**MITRAL INSUFFICIENCY**						
+	48 (48.5)	56 (52.3)	n.s.	46 (46.5)	58 (54.2)	n.s.
++	44 (44.4)	46 (43)	n.s.	45 (45.5)	45 (42.2)	n.s.
+++	7 (7.1)	5 (4.7)	n.s.	8 (8)	4 (3.6)	n.s.
**BIOMARKERS**						
LYMPHOCYTES	7.72 ± 2.18	7.64 ± 2.35	n.s.	7.74 ± 2.25	7.63 ± 2.51	n.s.
NEUTROPHILES	5.12 ± 1.80	5.16 ± 2.07	n.s.	5.16 ± 1.78	5.12 ± 2.21	n.s.
IL-6	24.03 ± 2.86	23.12 ± 2.56	n.s.	24.43 ± 2.72	23.02 ± 2.43	n.s.
CRP	8.96 ± 0. 89	8.15 ± 0.32	n.s.	8.93 ± 0. 90	8.45 ± 0.52	n.s.
BNP	182.95 ± 37.51	107.14 ± 33.25	0.0035^*^	175.41 ± 34.21	103.22 ± 31.25	0.004^*^
ST2	114.12 ± 5.1	57 ± 7.32	0.031^*^	111.38 ± 5.3	53 ± 7.12	0.004^*^
TnI	0.153 ± 0.01	0.156 ± 0.009	n.s.	0.152 ± 0.009	0.155 ± 0.008	n.s.
CKMB	1.221 ± 0.24	1.231 ± 0.21	n.s.	1.219 ± 0.23	1.243 ± 0.25	n.s.

In this table we have reported at 6 and 12th months of follow up clinical characteristics, drug therapy, and echocardiographic parameters comparing Metabolic Syndrome (MS) patients v/s overall population (no MS). ACE is for angiotensin converting enzyme; ARS is for angiotensin receptros; BMI is for body mass index; BNP is B type natriuretic peptide; CKMB is cretin kinase MB fraction; COPD is chronic obstructive pulmonary disease; CRP is C reactive protein; ICD-VVI is for single chamber internal cardioverter defibrillator; ICD-DDD is for dual chamber internal cardioverter defibrillator; IL-6 is interleukin 6; y is for year; in mitral insufficiency the simble + is for low grade of reflow, ++ for moderate grade of reflow, +++ is more than moderate; LVEDd is for left ventricle diastolic diameter; LVESd is for left ventricle systolic diameter; LVEDv is for left ventricle end diastolic volume; LVESv is for left ventricle end sistolic volume; LVEF is for left ventricle ejection fraction; n is for number; NYHA is for New York Hearth Association; NOAC is for new oral anticoagulant; SD is for standard deviation; ST2 is protein ST2; TnI is troponine I. Statistical analysis has been conducted to compare categorical data with the exact Pearson's X2 test. We considered a two-sided p-value of < 0.05 as statistically significant. A p-value < 0.05 has been marked with ^*^ symbol. A p > 0.05 is named as n.s.

**Figure 1:**
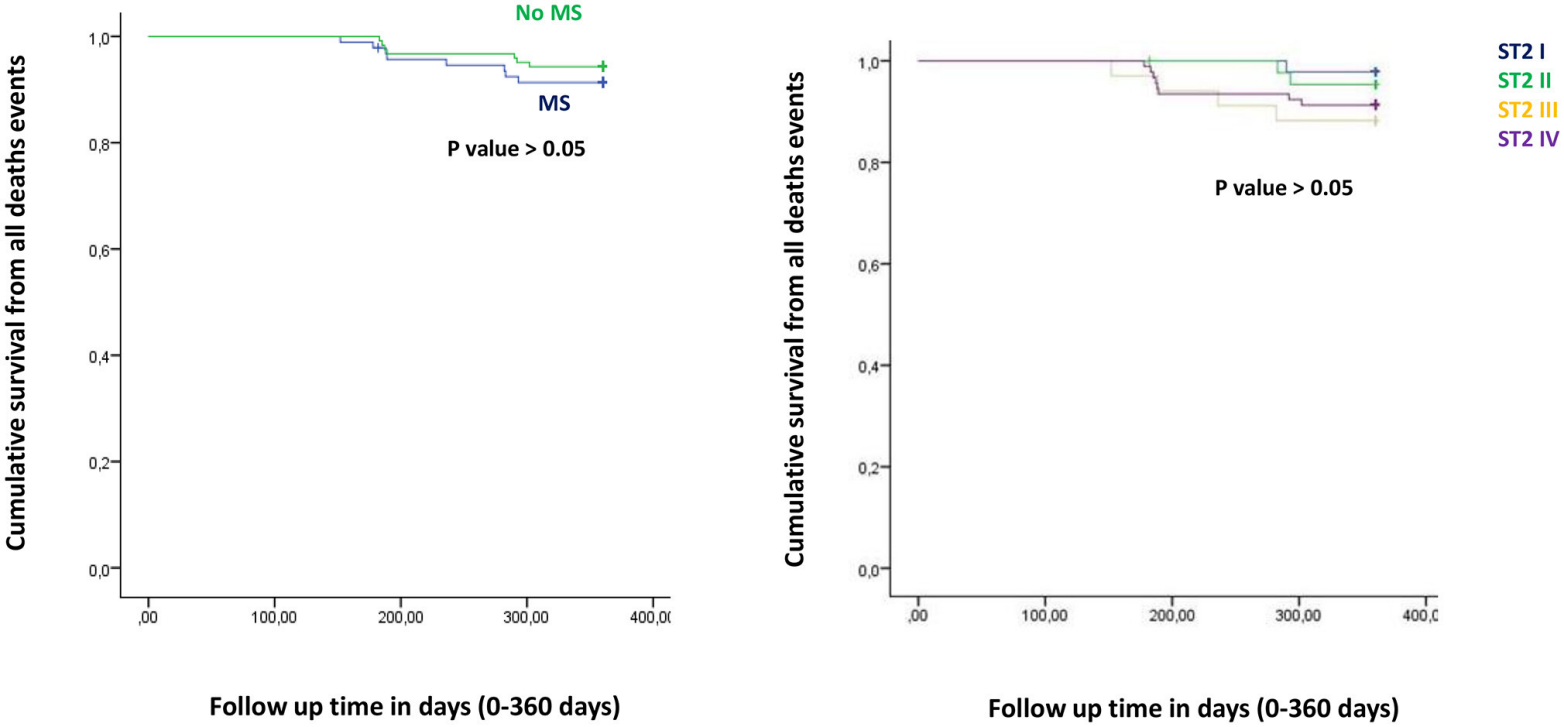
The curve representation of all deaths events as “cumulative survival from all deaths events” (on y axis) during 360 days follow up (on x axis) comparing MS vs. no MS patients (left part), and different quartiles (right part).

**Figure 2:**
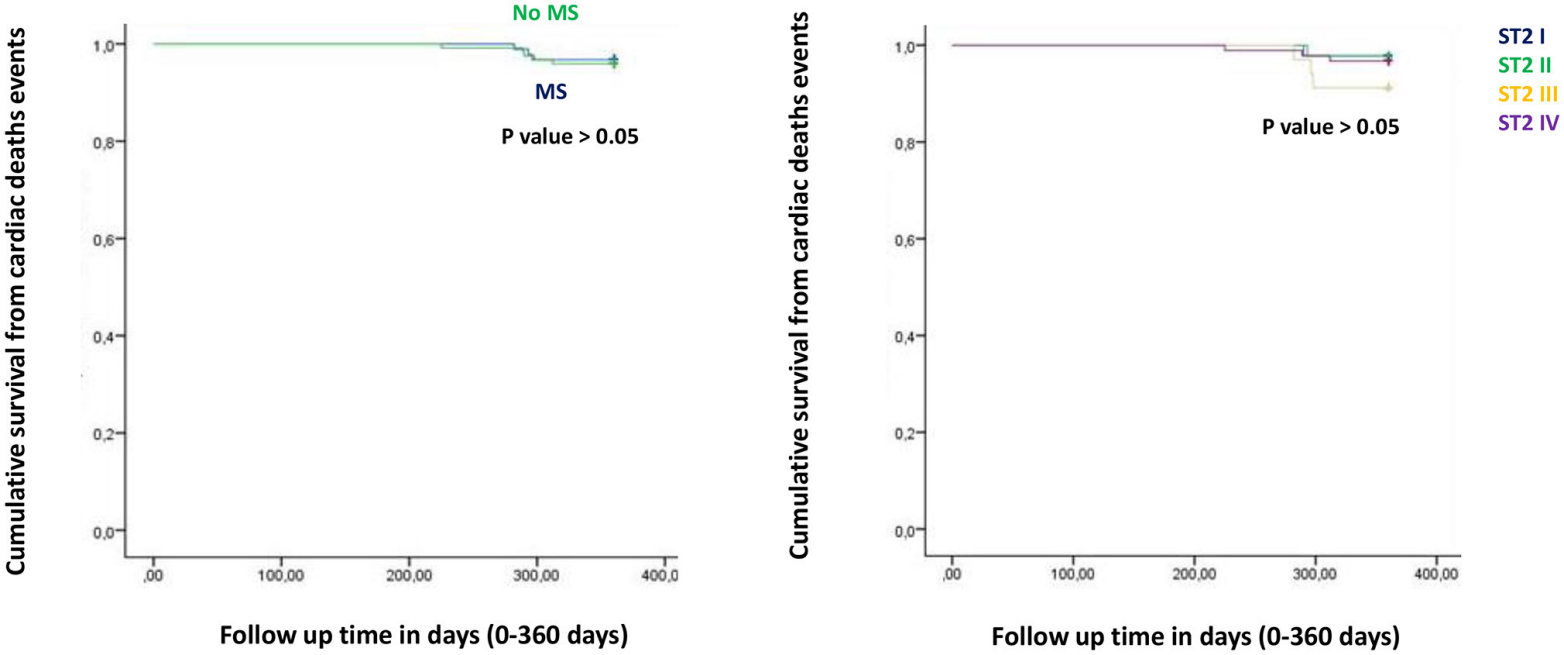
The curve representation of cardiac deaths events as “cumulative survival from cardiac deaths events” (on y axis) during 360 days follow up (on x axis) comparing MS vs. no MS patients (left part), and different quartiles (right part).

**Figure 3:**
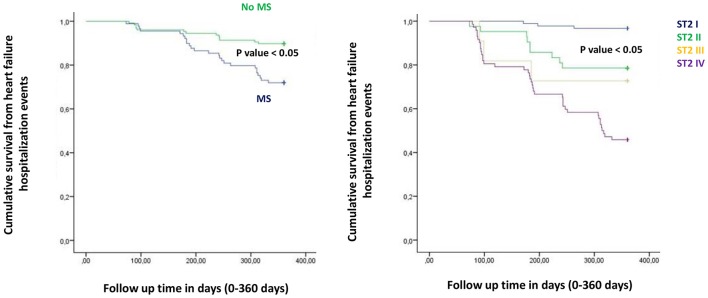
The curve representation of hospitalization for heart failure events as “cumulative survival from hospitalization for heart failure events” (on y axis) during 360 days follow up (on x axis) comparing MS vs. no MS patients (left part), and different quartiles (right part).

**Figure 4:**
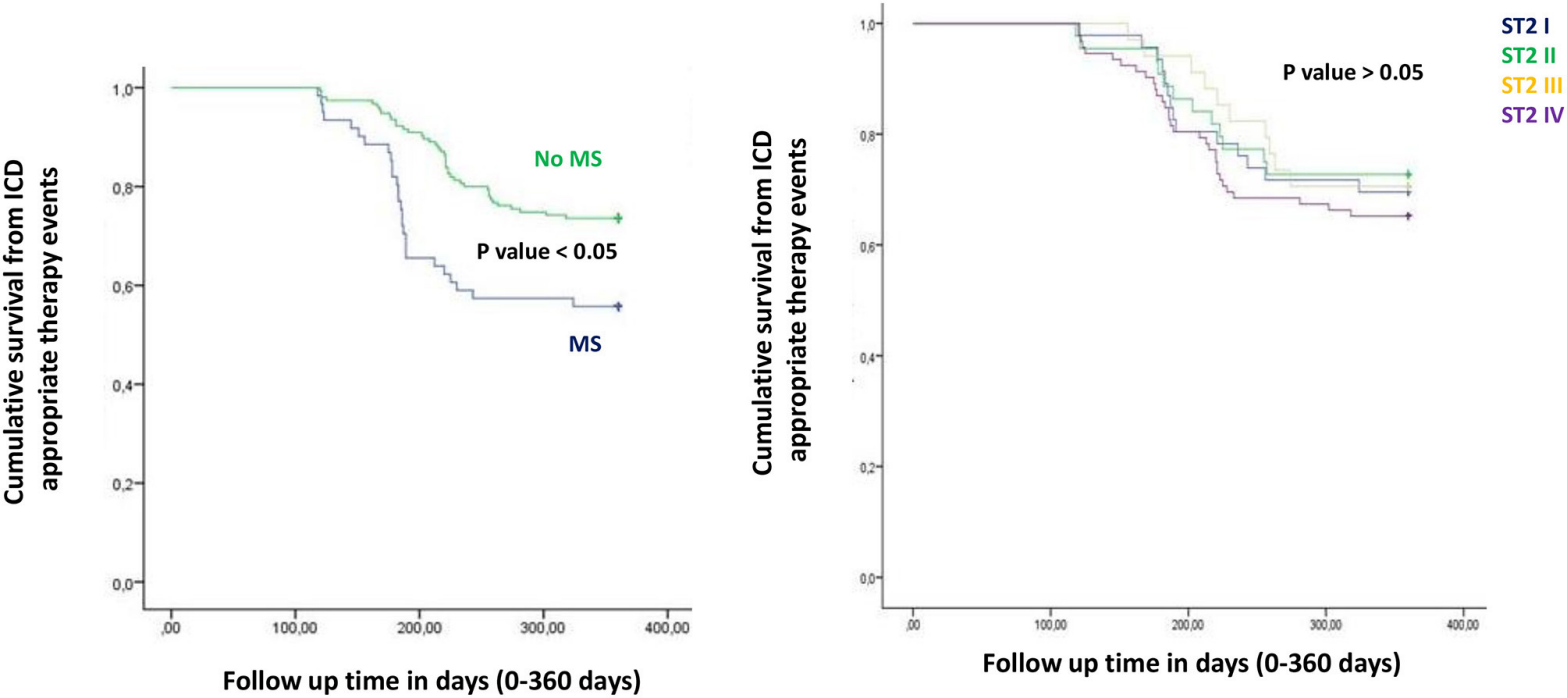
The curve representation of survival after ICD therapy events as “cumulative survival after ICD therapy events” (on y axis) during 360 days follow up (on x axis) comparing MS vs. no MS patients (left part), and different quartiles (right part).

**Figure 5:**
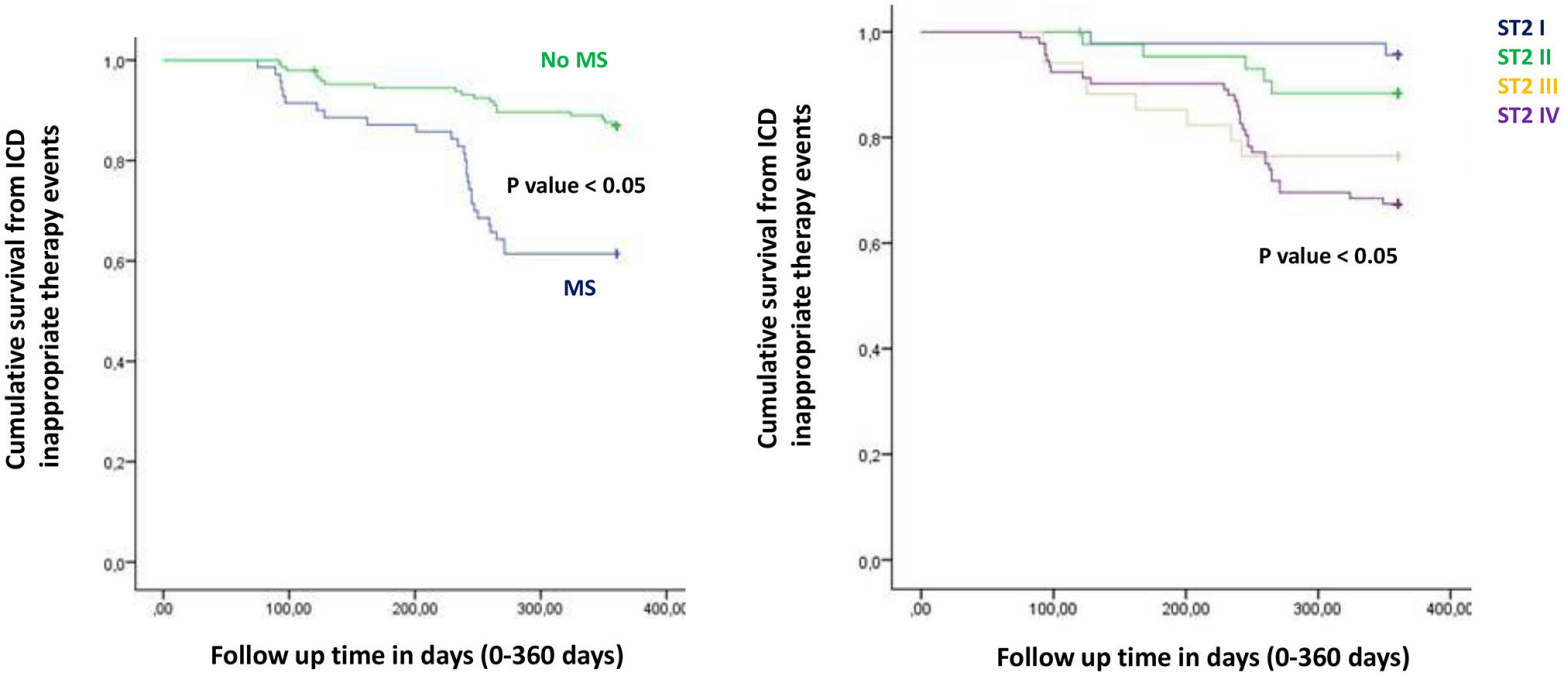
The curve representation of “cumulative survival from ICD appropriate therapy events” (on y axis) during 360 days follow up (on x axis) comparing MS vs. no MS patients (left part), and different quartiles (right part).

**Figure 6:**
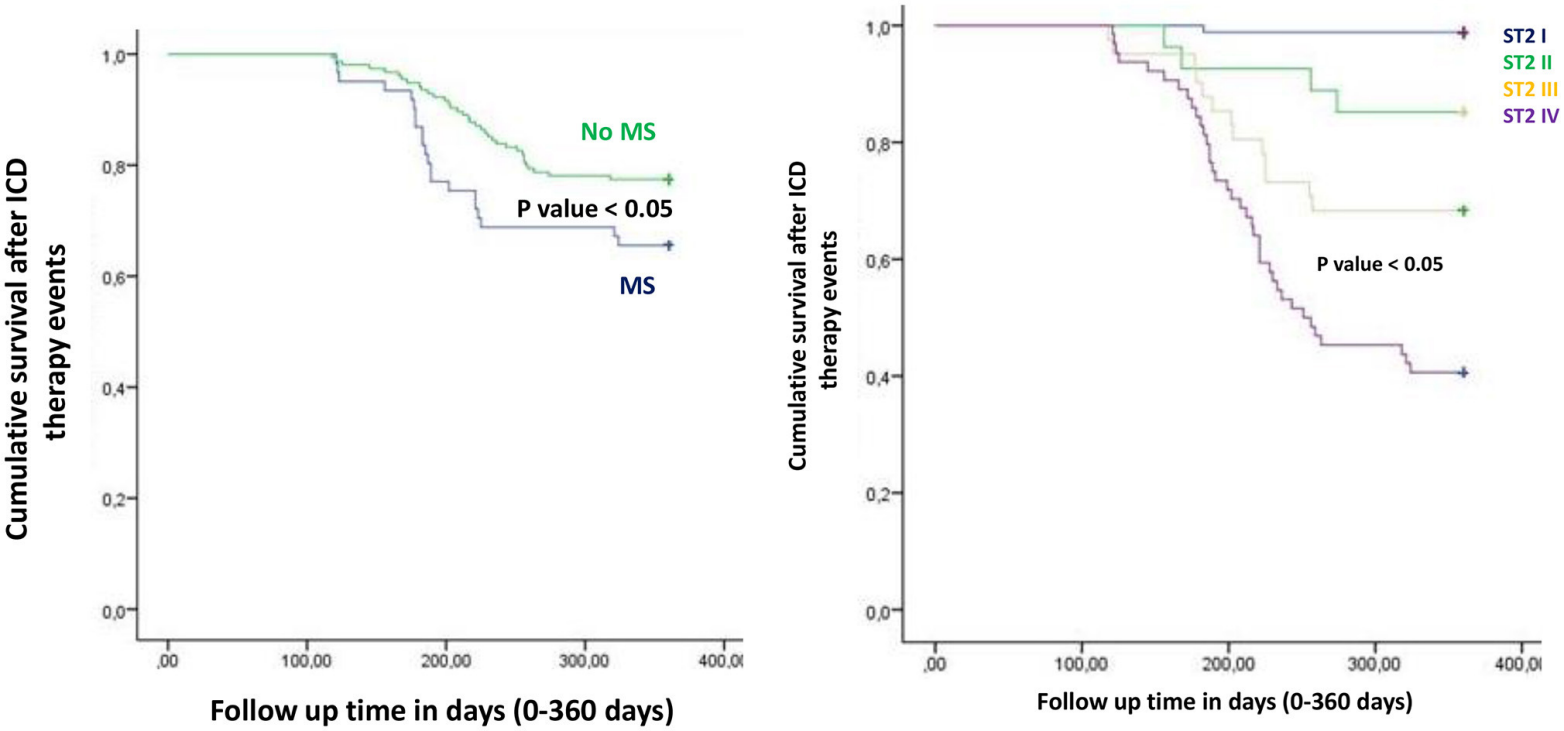
The curve representation of “cumulative survival from ICD inappropriate therapy events” (on y axis) during 360 days follow up (on x axis) comparing MS vs. no MS patients (left part), and different quartiles (right part).

**Figure 7:**
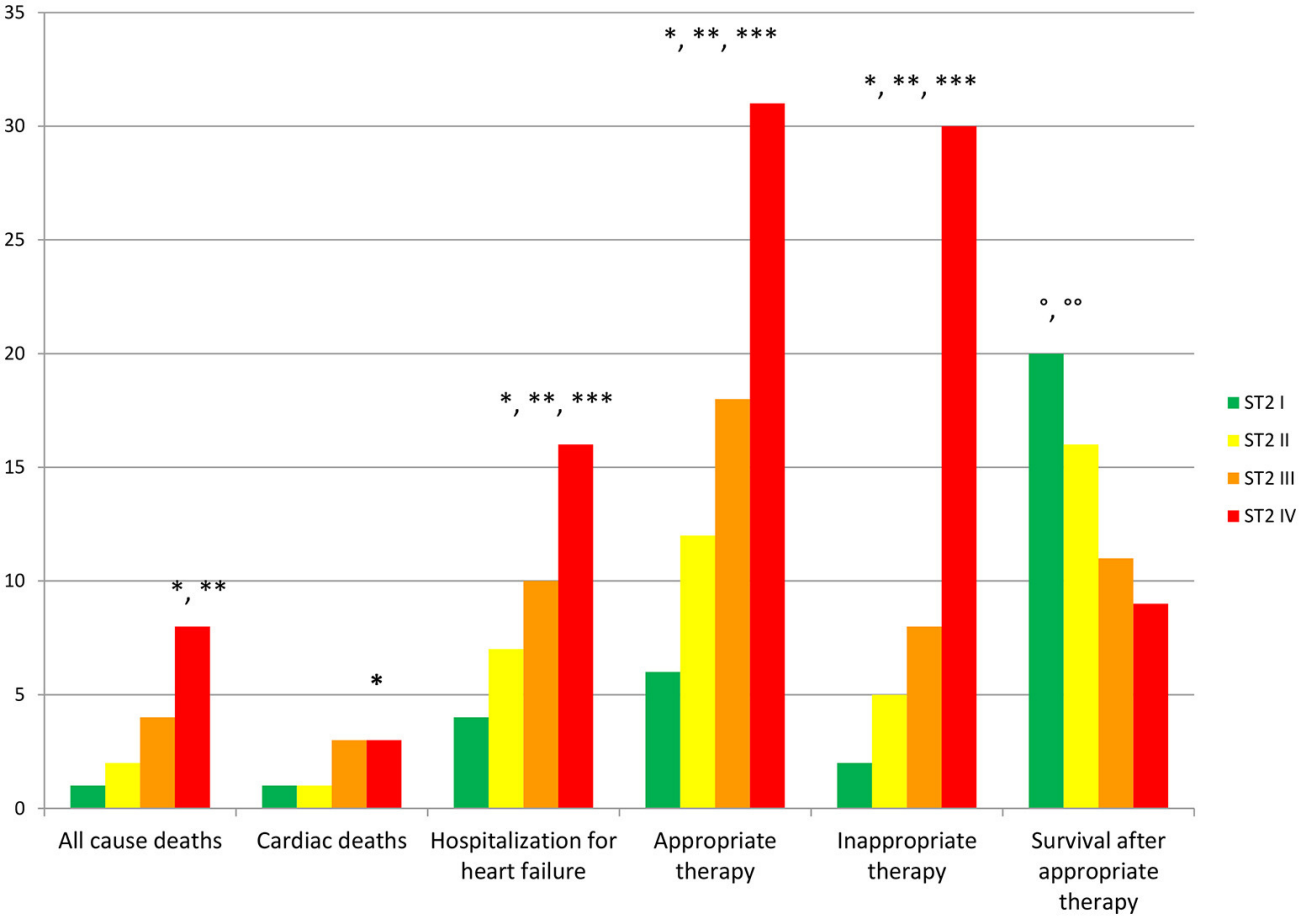
In the right part of the figure study outcomes reported for ST2 quartiles (I, II, III, IV quartile) in ng/ml. In the y axis number of events for each ST2 quartile. In the x axis the study outcomes. In green color I ST2 quartile. In yellow II ST2 quartile. In orange III ST2 quartile. In red IV ST2 quartile. A *p* < 0.05 is marked with symbol: ^*^ if ST2 IV vs. ST2 I; ^**^ if ST2 IV vs. ST2 III; ^***^ if ST2 IV vs. ST2 II; ° if ST2 I vs. ST2 IV; °° if ST2 I vs. ST2 III.

To date, at 6th and 12th month of follow up, MS vs. no MS patients reported higher rate of hospitalization for heart failure [24 (24.2%) vs. 13 (12.1%), *p*-value 0.04]. Table 3A. In addition, MS vs. no MS patients had higher rate of inappropriate therapy [27 (27.3%) vs. 18 (16.8%), *p*-value 0.05], and lower rate of appropriate therapy [26 (26.3%) vs. 41 (38.3%), *p*-value 0.05], and survival after appropriate therapy [21 (21.2%) vs. 35 (32.7%), *p*-value 0.05]. Table 3A. Intriguingly, stratifying the study outcomes for ST2 quartiles (number of events for each ST2 quartile), we reported that, highest ST2-values (IVth ST2 quartile) were associated with highest rate of all cause deaths (ST2 mean value 98.73 [23.75–203.73] ng/ml, *p* < 0.05), hospitalization for HF (ST2 mean value 140.83 [23.75–263] ng/ml, *p* < 0.05), appropriate therapy (ST2 mean value 128.92 [23.51–291.31] ng/ml, *p* < 0.05), and inappropriate therapy (ST2 mean value 166.60 [24.24–282] ng/ml, *p* < 0.05). Table 3B. The opposite trend was reported for Survival after appropriate ICD therapy. In fact, lowest ST2 values (Ith ST2 quartile) were associated with highest rate of survival after appropriate ICD therapy (30.68 [16.48–82.05] ng/ml, *p* < 0.05). Table 3B. At multivariate Cox regression analysis, CRP (HR 0.110 [0.027–0.446], 95% CI, *p*-value 0.002), TnI protein (HR 0.010 [0.001–0.051], 95% CI, *p*-value 0.010), and BNP blood values (HR 1.151 [1.010–1.510], 95% CI, *p*-value 0.001), were predictive for all cause of deaths. Table 4A. BNP blood values were predictive of cardiac deaths (HR 1.010 [1.001–1.206], 95% CI, *p*-value 0.033). Table 4B. MS, and BNP protein were both predictive of hospitalization for heart failure events (HR 2.902 [1.345–4.795], 95% CI, *p*-value 0.001; 1.005 [1.000–1.016], *p*-value 0.007). Table 4C. ST2 blood levels were predictive of appropriate therapy events (HR 1.012 [1.007–1.260], 95% CI, *p*-value 0.001), such as BNP (HR 1.005 [1.001–1.160], 95% CI, *p*-value 0.028), LVEF (HR 1.902 [1.857–1.950], 95% CI, *p*-value 0.001), and CRP (HR 1.833 [1.878–1.993], 95% CI, *p*-value 0.028). Table 4D. ST2, and BNP blood levels were predictive of inappropriate therapy events (HR 1.007 [1.003–1.011], 95% CI, *p*-value 0.002; HR 1.016 [1.001–1.091], 95% CI, *p*-value 0.012). Table 4E. At the end, ST2 and BNP blood values were predictive of survival after ICD appropriate therapy (HR 4.297 [1.985–9.302], 95% CI, *p*-value 0.001; HR 1.210 [1.072–1.685], 95% CI, *p*-value 0.024). Table 4F. All these outcomes in MS vs. no MS patients, and stratified for different ST2 quartiles were reported in Figures 1–**7**.

**Table 3:** Study endpoints.

**A**						
	**Study variables**					
**Study outcomes**	**MS (*****n*** **99)**	**No MS (*****n*** **107)**	***P*****-value**	**ICD-VVI (*****n*** **57)**	**ICD-DDD (*****n*** **151)**	***P*****-value**
All cause deaths (%)	8 (8.1)	7 (6.5)	n.s.	4 (7)	11 (7.2)	n.s.
Cardiac deaths (%)	3 (3.1)	5 (4.7)	n.s.	2 (3.5)	6 (3.9)	n.s.
Hospitalization for heart failure (%)	24 (24.2)	13 (12.1)	0.04^*^	11 (19.2)	26 (17.2)	n.s.
Appropriate therapy (%)	26 (26.3)	41 (38.3)	0.05^*^	20 (35)	47 (31.1)	n.s.
Inappropriate therapy (%)	27 (27.3)	18 (16.8)	0.05^*^	13 (22.8)	32 (21.2)	n.s.
Survival after appropriate therapy (%)	21 (21.2)	35 (32.7)	0.05^*^	17 (29.8)	39 (25.8)	n.s.
**B**						
	**Study variables**					
**Study outcomes**	**ST2 (mean value)**	**ST2 I**	**ST2 II**	**ST2 III**	**ST2 IV**	***P*****-value**
All cause deaths. n 15 (%)	98.73 [23.75-203.73]	1	2	4	8	< 0.05^*^. ^**^
Cardiac deaths. n 8 (%)	101.34 [23.75-203.73]	1	1	3	3	< 0.05^*^
Hospitalization for heart failure. n 37 (%)	140.83 [23.75-263]	4	7	10	16	< 0.05^*^. ^**^. ^***^
Appropriate therapy. n 67 (%)	128.92 [23.51-291.31]	6	12	18	31	< 0.05^*^. ^**^. ^***^
Inappropriate therapy. n 45 (%)	166.60 [24.24-282]	2	5	8	30	< 0.05^*^. ^**^. ^***^
Survival after appropriate therapy. n 56 (%)	30.68 [16.48-82.05]	20	16	11	9	< 0.05^°^. ^°°^

A. In this table we reported number of events of the study outcomes experienced in Metabolic Syndrome (MS) vs. controls (no MS), and in internal cardioverter defibrillator single chamber (ICD-VVI) vs. internal cardioverter defibrillator dual chambers (ICD-DDD). The symbol ^*^ marked a p < 0.05 comparing MS vs. no MS; the symbol ^**^ marked a p < 0.05 comparing ICD-VVI vs. ICD-DDD.

*B. In this table number of events of the study outcomes experienced in the ST2 protein quartiles (Ith, IIth, IIIth, and IVth quartile) in ng/ml (see the text). A p < 0.05 is marked with symbol*:

* *if ST2 IVth vs. ST2 Ith*;

** *if ST2 IVth vs. ST2 IIIth*;

*** *if ST2 IVth vs. ST2 IIth*;

° *if ST2 Ith vs. ST2 IVth*;

°° *if ST2 Ith vs. ST2 IIIth*.

**Table 4:** Cox regression analysis for study endpoints.

		**Univariate analysis**			**Multivariate analysis**	
	**HR**	**IC 95%**	***p*-value**	**HR**	**IC 95%**	***p*-value**
**(A) All cause of deaths**.						
MS	2.982	2.918–16.479	0.03	0.108	0.06–1.953	0.131
ST2	1.002	0.997–1.008	0.359	1.002	0.988–1.017	0.741
**BNP**	1.01	1.001–1.200	0.001	**1.151**	**1.010–1.510**	**0.001**
LVEF	0.873	0.793–0.960	0.005	0.994	0.874–1.131	0.928
**CRP**	0.601	0.394–0.916	0.018	**0.110**	**0.027–0.446**	**0.002**
IL-6	1.006	0.992–1.021	0.393	0.992	0.967–1.017	0.518
**TnI**	0.530	0.001–1.618	0.548	**0.010**	**0.001–0.051**	**0.010**
CKMB	0.970	0.122–7.693	0.977	1.841	0.040–10.3	0.754
NYHA III	3.660	1.838–7.287	0.029	0.001	0.001–5.25	0.889
NLr <2	0.951	0.377–2.395	0.915	0.070	0.010–0.489	0.007
**(B) Cardiac deaths**.						
MS	3.189	0.916–6.479	0.061	0.815	0.029–22.716	0.904
ST2	0.776	0.657–0.916	0.003	1.000	0.977–1.024	0.973
**BNP**	1.003	0.995–1.010	0.464	**1.010**	**1.001–1.206**	**0.033**
LVEF	0.808	0.627–1.041	0.099	0.986	0.80–1.216	0.897
CRP	1.001	1.000–1.01	0.001	0.442	0.125–1.558	0.204
IL−6	1.002	0.980–1.025	0.851	1.029	0.986–1.073	0.191
TnI	1.82	0.001–7.731	0.328	0.001	0.010–8.906	0.768
CKMB	0.761	0.040–14.417	0.855	0.230	0.010–46.54	0.587
NYHA III	3.092	0.248–3.85	0.115	0.010	0.001–4.161	0.943
NLr <2	1.187	0.319–4.421	0.798	3.305	0.231–47.358	0.379
**(C) Hospitalization for heart failure**.						
**MS**	1.051	1.018–1.140	0.001	**2.902**	**1.345–4.795**	**0.001**
ST2	0.978	0.931–1.029	0.393	1.002	0.997–1.007	0.349
**BNP**	1.008	1.005–1.011	0.001	**1.005**	**1.000–1.016**	**0.007**
LVEF	0.942	0.900–0.986	0.10	0.970	0.919–1.025	0.286
CRP	1.02	1.05–1.420	0.001	0.963	0.890–1.042	0.351
IL-6	0.999	0.990–1.010	0.885	1.006	0.995–1.017	0.285
TnI	1.692	0.001–2.210	0.978	0.051	0.001–24.92	0.084
CKMB	0.756	0.226–2.522	0.649	0.246	0.039–1.559	0.136
NYHA III	7.305	4.134–12.911	0.001	0.471	0.175–1.270	0.137
NLr <2	1.165	0.682–1.989	0.576	0.803	0.417–1.549	0.514
**(D) Appropriate therapy**.						
MS	1.058	1.023–1.146	0.001	6.432	0.56–16.156	0.150
**ST2**	0.981	0.932–1.032	0.462	**1.012**	**1.007–1.260**	**0.001**
**BNP**	1.008	1.005–1.011	0.001	**1.005**	**1.001–1.160**	**0.028**
**LVEF**	0.942	0.900–0.986	0.010	**1.902**	**1.857–1.950**	**0.001**
**CRP**	1.01	1.000–1.130	0.001	**1.833**	**1.678–1.993**	**0.028**
IL−6	0.989	0.980–1.009	0.885	0.997	0.998–1.006	0.474
TnI	1.692	0.001–2.010	0.097	0.031	0.001–16.193	0.197
CKMB	0.756	0.226–2.522	0.649	0.537	0.158–1.819	0.318
NYHA III	7.305	4.134–12.911	0.001	0.829	0.324–2.122	0.695
NLr <2	1.165	0.682–1.989	0.576	0.751	0.452–1.247	0.269
**(E) Inappropriate therapy**.						
MS	1.085	1.033–1.214	0.001	0.963	0.773–7.879	0.063
**ST2**	1.003	0.949–1.060	0.920	**1.007**	**1.003–1.011**	**0.002**
**BNP**	1.007	1.003–1.010	0.001	**1.016**	**1.001–1.091**	**0.012**
LVEF	0.984	0.950–1.020	0.384	0.991	0.934–1.046	0.742
CRP	1.008	1.002–1.712	0.001	1.026	0.980–1.074	0.281
IL−6	1.010	1.001–1.019	0.032	1.003	0.993–1.013	0.551
TnI	0.010	0.002–8.261	0.063	0.510	0.010–16.813	0.291
CKMB	1.093	0.299–3.990	0.893	0.657	0.149–2.896	0.579
NYHA III	1.667	0.918–3.027	0.93	0.916	0.348–2.412	0.859
NLr <2	1.109	0.617–1.991	0.730	1.520	0.816–2.832	0.187
**(F) Survival after appropriate therapy**.						
MS	6.252	3.998–9.778	0.001	1.001	0.997–1.005	0.494
**ST2**	0.985	0.954–1.018	0.370	**4.297**	**1.985–9.302**	**0.001**
**BNP**	0.977	0.971–0.983	0.001	**1.210**	**1.072–1.685**	**0.024**
LVEF	1.016	0.996–1.036	0.111	0.972	0.923–1.023	0.270
CRP	1.21	1.002–1.531	0.001	0.960	0.913–1.010	0.112
IL-6	1.001	0.994–1.007	0.821	1.002	0.994–1.011	0.750
TnI	1.41	0.002–7.128	0.448	2.925	0.010–7.389	0.691
CKMB	1.774	0.807–3.896	0.154	1.408	0.404–4.913	0.591
NYHA III	0.725	0.483–1.089	0.121	1.367	0.578–3.233	0.476
NLr <2	1.162	0.818–1.651	0.402	1.219	0.706–2.104	0.478

*In this table the representation of study outcomes, as all cause of deaths (a), cardiac deaths (b), hospitalization for heart failure (c), appropriate therapy (d), inappropriate therapy (e), and survival after appropriate therapy (f), and multivariate predictive factors. BNP is for B type natriuretic peptide; CKMB is creatine kinase MB type; CRP is C reactive protein; IL-6 is interleukin 6; LVEF is left ventricle ejection fraction; MS is Metabolic syndrome; NYHA III is New York Heart Association third class; NLr <2 is indicating a neutrophiles/lymphocytes ratio <2; ST2 is protein ST2; TnI is troponine I. We have used for statistical analysis, a 95% interval of confidence (CI), and a significant statistical p-value, p <0.05. To test the final statistical used model, we have performed the Hosmer and Lemeshow test, with a χ^2^ = 2.775, and a p < 0.05. The bold values mean that statistical significant p < 0.05*.

Figure from Figures 1–**6** the representation of cumulative survival events free curves for study endpoints, by Cox regression analysis in Metabolic Syndrome (MS) (blue color) vs. no MS patients (green color) in the left part, and in different ST2 quartiles (ST2 first quartile (I) in blue color, ST2 second (II) quartile in green color, ST2 third (III) quartile in yellow color, and ST2 fourth (IV) quartile in violet color), in the right part. In the **Figure 7** the representation of study outcomes stratified for ST2 quartiles. The statistical significant event is indicated by a *p* < 0.05.

## Discussion

In the present study MS patients vs. no MS patients showed higher serum levels of inflammatory, and myocardial stress biomarkers at baseline. Excluding the inflammatory tone, this trend was maintained at 6th and 12th month follow up. Therefore, in MS patients we reported a constant, and gradually altered synthesis, and peripheral relapse of BNP, and ST2 protein, that are two well known peptides and markers of myocardial stress (Pascual-Figal and Januzzi, 2015). Conversely, these alterations caused modifications of the ionic channels function, and of cardiac structure, leading to a pro-arrhyhtmic status in MS patients (Sardu et al., 2017). From our results, CRP (HR 0.110 [0.027–0.446], 95% CI, *p*-value 0.002), TnI protein (HR 0.010 [0.001–0.051], 95% CI, *p*-value 0.010), and BNP blood values (HR 1.151 [1.010–1.510], 95% CI, *p*-value 0.001), were all predictive for all cause of deaths. CRP and TnI are markers of atherosclerotic plaque inflammation, and augmented cardiac injury during acute coronary syndrome, and then related to worse prognosis (January et al., 2014). In heart failure patients these biomarkers reflected the hyper activity of inflammatory tone, and of the cardiac injury, then associated to worse cardiovascular function, and higher rate of all cause of deaths (Braunwald, 2008). To date, BNP may be an independent predictor all cause deaths (HR 1.151 [1.010–1.510], 95% CI, *p*-value 0.001). This study result is in line with different studies, indicating BNP as a strong prognostic predictor for all cause of deaths in asymptomatic patients, and in patients with heart failure at all stages of disease (Doust et al., 2004, 2005; Braunwald, 2008; Scott et al., 2011). In fact, BNP is released by the heart in condition of hypertrophy, mechanical stress, oxidative stress, and in response to augmented myocardial tension, and increased intravascular volume (Doust et al., 2004, 2005; Braunwald, 2008). Therefore, BNP is routinely used for the diagnosis and the monitoring of heart failure (Braunwald, 2008), and is a consistent significant prognostic indicator of deaths in failing heart patients (Lee et al., 2003; Doust et al., 2004, 2005; Braunwald, 2008; Usuku et al., 2010). Conversely, in our study BNP predicted cardiac deaths (HR 1.010 [1.001–1.206], 95% CI, *p*-value 0.033), and hospitalization for heart failure worsening (HR 1.005 [1.000–1.016], 95% CI, *p*-value 0.007), such as MS disease (HR 2.902 [1.345–4.795], 95% CI, *p*-value 0.001). Table 4C. The BNP has been broadly proposed as a predictor of mortality event in HF patients, by a direct correlation of its blood values with cardiac pump failure, and also as independent predictor of pump failure in ischemic, and non-ischemic HF patients (Lee et al., 2003; Doust et al., 2004, 2005; Braunwald, 2008; Usuku et al., 2010). The MS enhances the entity of myocardial inflammation, myocardial stretch and fibrosis, and subsequent cardiac damage (Braunwald, 2008; Sardu et al., 2016). The MS pro-arrhythmic status causes electrical and anatomical systolic and diastolic cardiac alterations, leading to the failure of cardiac pump, and subsequently affecting ICDs' leads parameters functionality, and the related outcomes in failing heart patients (Sardu et al., 2017). In our study these alterations may lead to a higher rate of hospitalization for heart failure in MS vs. no MS patients [24 (24.3%) vs. 13 (12.1%), *p*-value 0.04]. Conversely, we reported a statistical significant higher rate of survival free from heart failure hospitalizations in patients with lowest ST2 values (I quartile) as compared to other ST2 quartiles. Figure 3. This may also affect the higher rate of inappropriate therapy [27 (27.3) vs. 18 (16.8), *p*-value 0.05], and appropriate therapy events [26 (26.3) vs. 41 (38.3), *p*-value 0.05], and the lower rate of survival after appropriate therapy [21 (21.2) vs. 41 (38.3), *p*-value 0.05], in MS vs. no MS patients treated by an ICD. Table 3A, Figures 4–**6**. However, stratifying these clinical outcomes for ST2 quartiles, highest baseline ST2 values were linked to worse prognosis, such as all cause of deaths (ST2 mean value 98.73 [23.75–203.73] ng/ml, *p* < 0.05), higher rate of hospitalization for HF (ST2 mean value 140.83 [23.75–263] ng/ml, *p* < 0.05), appropriate therapy (ST2 mean value 128.92 ± [23.51–291.31] ng/ml, *p* < 0.05), and inappropriate therapy events (ST2 mean value 166.60 ± [24.24–282] ng/ml, *p* < 0.05). Table 3B, **Figure 7**. On the other hand, the opposite trend was reported for survival rate after appropriate ICD therapy events. In this case, lowest ST2 values (30.68 ± [16.48–82.05] ng/ml, *p* < 0.05) were associated to the higher percentage of survived patients. Table 3B, Figures 5, **7**. To date, in survival curve the lowest ST2 values (ST2 I quartile) were associated to higher rate of survival after ICD intervention. Figure 4. Therefore, we may speculate that, lowest ST2 values may be linked to a lower degree of cardiac fibrosis, and of electro-anatomical alterations in failing heart MS patients. These alterations may result in a lower rate of arrhythmias, due to a more efficient cardiac electrical and mechanical function, and consequently leading to a more favorable cardiac pump function. This may reduce the arrhythmic burden, and subsequently the ICDs' interventions, leading to a better survival rate. For all these processes, and clinical outcomes, we have to recognize the importance of ST2 protein assay in failing heart patients affected by MS, as a monitoring and predictive biomarker in stable chronic HF patients treated by ICD. ST2 protein is a member of the interleukin 1 receptor family (Pascual-Figal and Januzzi, 2015; Priori et al., 2015), expressed and relapsed by cardiac cells in response to myocardial stress (Ky et al., 2011). ST2 protein by the interaction with the trans membrane receptor ST2L isoform reduced myocardial fibrosis, cardiomyocyte hypertrophy, and apoptosis, improving myocardial function (Ky et al., 2011). In HF patients this homeostatic balance between synthesis, relapse, and function of ST2 is lost, and the IL-33/ ST2 system is up-regulated in cardiomyocytes, and fibroblasts in response to acute cardiac injury, and during chronic adaptive condition of pump failure such as heart failure (Tominaga, 1989; Alpert et al., 2000; Weinberg et al., 2002; Braunwald, 2008; Ky et al., 2011). Therefore, to the opposite face of the coin, the higher serum levels of ST2 protein may be associated to advanced cardiac fibrosis, leading to a pro-arrhythmic status, and to diastolic and systolic cardiac alterations, conditioning an irreversible cardiac pump failure. This may be a factor leading to a worse prognosis in HF patients. Regards appropriate therapy events, these events may be predicted by ST2 (HR 1.012 [1.007–1.260], 95% CI, *p*-value 0.001), BNP (HR 1.005 [1.001–1.160], 95% CI, *p*-value 0.028), LVEF (HR 1.902 [1.857–1.950], 95% CI, *p*-value 0.001), and CRP serum levels (HR 1.833 [1.878–1.993], 95% CI, *p*-value 0.028). Table 4D. These points were broadly discussed by authors in a population of ICDs' patients with advanced systolic left ventricle dysfunction (Scott et al., 2011). Actually, we evaluated similar findings in a population of MS failing heart patients, suggesting that, all these biomarkers tested by peripheral blood assay, may accurately characterize heart failure severity, disease stage, and to predict clinical adverse clinical events. Intriguingly, elevated ST2 and BNP values may predict also inappropriate ICD therapy (HR 1.007 [1.003–1.011], 95% CI, *p*-value 0.002; HR 1.016 [1.001–1.091], 95% CI, *p*-value 0.012). Table 4E. Consequently, ST2, and BNP blood values may predict the survival rate after ICD appropriate therapy (HR 4.297 [1.985–9.302], 95% CI, *p*-value 0.001; HR 1.210 [1.072–1.685], 95% CI, *p*-value 0.024) in our study population. Table 4F. However, ST2 and BNP together represent two myocardium stretching peptides useful to monitor HF disease stage, and to predict ICD interventions. These study result underline the importance of ST2 protein evaluation, added to BNP measurements in failing heart MS patients treated by ICD. These study results may be graphically seen in Figures 1–6, in that we reported study outcomes for MS vs. no MS patients, and differentially for each ST2 quartile (I-IV). It is interesting to observe, that there is a higher statistical significant cumulative survival after ICD appropriate therapy in ST2 first quartile, as compared to other ST2 quartiles (*p* < 0.05). Figures 4, 7. We might take in consideration this study result as a new information in the field of MS patients treated by ICD. In fact, in the setting of the inflammatory, oxidative, and electrophysiological alterations enhanced by the MS condition (Sardu et al., 2017), over the BNP also the ST2 protein may be a new HF relevant biomarker measurable in ICDs' recipients. In our opinion, ST2 routinely evaluation may represent in HF disease field the opportunity to add new informations bridging the inflammatory axis with myocardial wall stretching, and myocardial fibrosis, and crossing for cardiomyocyte hypertrophy, and apoptosis, that are adaptive HF processes leading to alterations of the myocardial function (Ky et al., 2011; Bayes-Genis et al., 2013; Pascual-Figal and Januzzi, 2015). To date, ST2 protein and its two main isoforms, as transmembrane or cellular (ST2L) and soluble or circulating (sST2) forms (Ky et al., 2011; Pascual-Figal and Januzzi, 2015), and the IL-33/ ST2 system are up regulated in cardiomyocytes and fibroblasts in response to cardiac injury, and in patients with chronic HF (Ky et al., 2011; Bayes-Genis et al., 2013). Moreover, a single ambulatory measurement of sST2 may be independently associated with adverse outcomes in failing heart subjects, because it is linked with functional capacity and long-term clinical outcomes (Felker et al., 2013). These results were confirmed in the Valsartan Heart Failure trial (Anand et al., 2014), and by Gruson et al in heart failure patients with reduced LVEF as the strongest predictor of cardiovascular death (Gruson et al., 2014). Not far from here, also the MUSIC trial reported that, elevation of ST2 and NT-proBNP above the cut-off value were associated with a high rate of sudden death (71%), in contrast with a very low rate (4%) when the two biomarkers were below the threshold (Pascual-Figal et al., 2009). We may speculate that, these uncontrolled and unbalanced alterations may reduce the functionality of cardiac pump, increasing the risk of ventricular arrhythmias, and subsequent sudden cardiac deaths events (SCD). Moreover, the routine clinical application of ST2 assay in HF patients is supported by all these clinical trial (Pascual-Figal et al., 2009; Felker et al., 2013; Anand et al., 2014; Gruson et al., 2014). In other words the ST2 values are due to a fine cross talking between wall stress, inflammation, fibrosis, as well as other numerous and not well known inputs (Braunwald, 2008; Pascual-Figal et al., 2009; Anand et al., 2014; Gruson et al., 2014). All these processes may be differently expressed and correlated to worse prognosis in HF patients, and in failing heart MS vs. no MS patients.

## Conclusions

Serum levels of BNP and ST2 protein may differentiate higher risk patients to experience ICDs' therapy, and worse prognosis. Highest ST2 values may be linked to a major rate of hospitalization, and ICDs' therapies, while on the contrary lowest ST2 values may be predictive of higher rate of survival after ICD appropriate therapy. Intriguingly, lowest ST2 values may predict a four times greater rate of survival after appropriate ICD therapy (HR 4.297 [1.985–9.302], CI 95%, *p*-value 0.001). In this setting, ST2 protein is a unique marker of myocardial stress, neuro-hormonal axis dysfunction, and sympathetic hyper activation in heart failure patients (Yancy et al., 2013). To date, the ST2 complex function bridging between all these alterations, and cross talking with the neuro-hormonal activation in heart failure patients, may represent the key protein for all these adaptive processes in MS vs. no MS patients. Therefore, ST2 protein may be used as an attractive diagnostic, and prognostic HF biomarker, such as a serum protein to monitor the response to ICD therapy in MS patients. However, all these different mechanisms ST2 linked have to be fully investigated, and more in detail elucidated in future research trials in MS patients. In the future ST2 protein may become an attractive therapeutic target to reduce ICD inappropriate therapy, and to improve the survival in ICDs' patients affected by MS. To date, ICD therapy and survival in MS patients implanted for primary prevention is still not a predictable event, and the benefit of ST2 evaluation in addition to other cardiac biomarkers and currently available clinical risk prediction models is still unclear. Therefore, these results demand a confirmatory prospective cohort study, designed and powered to derive and validate prediction algorithms incorporating these markers.

## Study limitation

In this prospective multicenter study we have examined a small percentage of MS patients treated by ICD, as compared to overall population. This is due to loss of patients during follow up, and to the low adherence of patients to the study protocol as discussed in results session. This study has been conducted at 12 months follow up time, and this short time follow up duration may affect the long term follow up prognosis and primary and secondary clinical outcomes. We have to report the paucity of clinical characteristics, that would provide a more accurate comparison to clinical trial subjects. At last we have not investigated the molecular, and epigenetic aspects related to heart failure patients, and/or modulated by interventional treatments (Marfella et al., 2013; Sardu et al., 2014). We do not have an animal model to compare in experiment setting these study results from humans. We do not have for all patients imaging data to evaluate deeply scar extension in all patients.

## Author contributions

CeS wrote the research protcol, and study design. RM and GP revised the manuscript before to submit. MS, QP, and CeS performed ICD implants. SP performed laboratory analysis. DC perfomred echocardiographic assessment. MR and MB revised the manuscript. This study has been conducted without sponsors and without financial support. CeS has written the study protocol, collected, analyzed data written the article. All authors have reviewed and approved the text before to submit the manuscript.

### Conflict of interest statement

The authors declare that the research was conducted in the absence of any commercial or financial relationships that could be construed as a potential conflict of interest. The reviewer MC and handling Editor declared their shared affiliation.

## Abbreviations

ATP: anti-tachycardia pacing
BNP: B-type natriuretic peptide
CAD: coronary artery disease
CI: confidence interval
CKMB: creatin kinase MB isoform
CRP: C-reactive protein
HF: heart failure
ICD: implantable cardioverter-defibrillator
ICD VVI: single chamber implantable cardioverter-defibrillator
ICD DDD: dual chambers implantable cardioverter-defibrillator
LVEF: left ventricle ejection fraction
NICM: non-ischemic dilated cardiomyopathy
NYHA: New York Heart Association class
NLr: neutrophiles-lymphocytes ratio
SCD: sudden cardiac death
ST2: ST2 protein
TC: total cholesterol
TnI: troponine I isoform
VT: ventricular tachycardia
VF: ventricular fibrillation.
